# (1*S*,5*R*)-1-(3,4-Dichloro­phen­yl)-3-oxa­bicyclo­[3.1.0]hexan-2-one

**DOI:** 10.1107/S1600536809007533

**Published:** 2009-03-06

**Authors:** Carl Henrik Görbitz, Tore Hansen, Kristian Vestli

**Affiliations:** a Department of Chemistry, University of Oslo, P.O.Box 1033 Blindern, N-0315 Oslo, Norway

## Abstract

The absolute structure has been determined by X-ray analysis for the title compound, C_11_H_8_Cl_2_O_2_. The five-membered ring of the mol­ecule is best described as a flattened envelope conformation with the methyl­ene C atom located 0.208 (2) Å below the plane formed by the other four atoms. A weak intermolecular C—H⋯O hydrogen bond is present in the crystal structure

## Related literature

The title compound was prepared as an inter­mediate in the search for potential triple neurotransmittor reuptake inhibitors, see: Milewska *et al.* (1996 ▶); Lin & Charette (2005 ▶); Tsuji *et al.* (1999 ▶); Džolić *et al.* (2003 ▶).
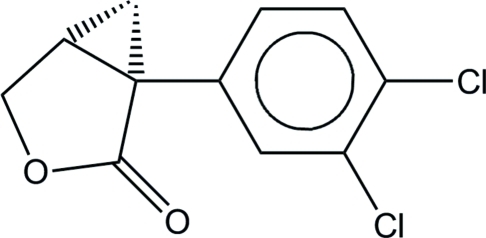


## Experimental

### 

#### Crystal data

C_11_H_8_Cl_2_O_2_
*M* *_r_* = 243.07
Orthorhombic, 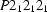

*a* = 7.0597 (4) Å
*b* = 11.1343 (7) Å
*c* = 12.6756 (8) Å
*V* = 996.36 (11) Å^3^
*Z* = 4
Mo *K*α radiation
μ = 0.62 mm^−1^
*T* = 102 K
0.58 × 0.36 × 0.18 mm

#### Data collection

Bruker APEXII CCD diffractometer
Absorption correction: multi-scan (*SADABS*; Sheldrick, 1996 ▶) *T*_min_ = 0.680, *T*_max_ = 0.894
8562 measured reflections
2341 independent reflections
2278 reflections with *I* > 2σ(*I*)
*R*_int_ = 0.018

#### Refinement

*R*[*F* ^2^ > 2σ(*F* ^2^)] = 0.024
*wR*(*F* ^2^) = 0.063
*S* = 1.10
2341 reflections
160 parameters
Only H-atom coordinates refined
Δρ_max_ = 0.43 e Å^−3^
Δρ_min_ = −0.20 e Å^−3^
Absolute structure: Flack (1983 ▶), 952 Friedel pairs
Flack parameter: 0.04 (5)

### 

Data collection: *APEX2* (Bruker, 2007 ▶); cell refinement: *SAINT-Plus* (Bruker, 2007 ▶); data reduction: *SAINT-Plus*; program(s) used to solve structure: *SHELXTL* (Sheldrick, 2008 ▶); program(s) used to refine structure: *SHELXTL*; molecular graphics: *SHELXTL*; software used to prepare material for publication: *SHELXTL*.

## Supplementary Material

Crystal structure: contains datablocks I, global. DOI: 10.1107/S1600536809007533/xu2480sup1.cif

Structure factors: contains datablocks I. DOI: 10.1107/S1600536809007533/xu2480Isup2.hkl

Additional supplementary materials:  crystallographic information; 3D view; checkCIF report

## Figures and Tables

**Table 1:** Hydrogen-bond geometry (Å, °)

*D*—H⋯*A*	*D*—H	H⋯*A*	*D*⋯*A*	*D*—H⋯*A*
C7—H71⋯O2^i^	0.880 (18)	2.366 (18)	3.2443 (16)	175.6 (16)

Symmetry code: (i) 
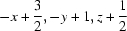
.

**Fig. 1.**
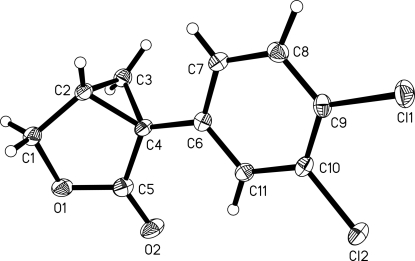
The molecular structure of the title compound. Displacement ellipsoids are shown at the 50% probability level and H atoms are shown as spheres of arbitrary size.

## supplementary crystallographic information

### Comment

The title compound was prepared as an intermediate in the search for potential triple neurotransmittor reuptake inhibitors (Milewska *et al.*, 1996; Lin & Charette, 2005; Tsuji *et al.*, 1999; Džolić *et al.*, 2003); details will be published elsewhere.

The molecular structure is shown in Fig. 1. The five-membered ring of the molecule is best described as a flat envelope conformation with C1 located 0.208 (2) Å below the plane constituted by C2, C4, C5 and O1, on the opposite side of C3. In the crystal structure the weak C—H···O hydrogen bonding presents between benzene ring and the carbonyl O atom of the neighboring molecule (Table 1).

### Experimental

Block-shaped single crystals were obtained from an acetonitrile solution by slow evaporation at room temperature.

### Refinement

Positional parameters were refined for all H atoms, *U*_iso_(H) values were set to 1.2*U*_eq_(C).

### Crystal data

**Table:**

C_11_H_8_Cl_2_O_2_	*D*_x_ = 1.620 Mg m^−^^3^
*M**_r_* = 243.07	Mo *K*α radiation, λ = 0.71073 Å
Orthorhombic, *P*2_1_2_1_2_1_	Cell parameters from 6286 reflections
*a* = 7.0597 (4) Å	θ = 2.4–27.9°
*b* = 11.1343 (7) Å	µ = 0.62 mm^−^^1^
*c* = 12.6756 (8) Å	*T* = 102 K
*V* = 996.36 (11) Å^3^	Block, colourless
*Z* = 4	0.58 × 0.36 × 0.18 mm
*F*(000) = 496	

### Data collection

**Table:**

Bruker APEXII CCD diffractometer	2341 independent reflections
Radiation source: fine-focus sealed tube	2278 reflections with *I* > 2σ(*I*)
graphite	*R*_int_ = 0.018
Detector resolution: 8.3 pixels mm^-1^	θ_max_ = 27.9°, θ_min_ = 2.4°
Sets of exposures each taken over 0.5° ω rotation scans	*h* = −9→8
Absorption correction: multi-scan (*SADABS*; Sheldrick, 1996)	*k* = −14→14
*T*_min_ = 0.680, *T*_max_ = 0.894	*l* = −16→16
8562 measured reflections	

### Refinement

**Table:**

Refinement on *F*^2^	Secondary atom site location: difference Fourier map
Least-squares matrix: full	Hydrogen site location: inferred from neighbouring sites
*R*[*F*^2^ > 2σ(*F*^2^)] = 0.024	Only H-atom coordinates refined
*wR*(*F*^2^) = 0.063	*w* = 1/[σ^2^(*F*_o_^2^) + (0.0387*P*)^2^ + 0.112*P*] where *P* = (*F*_o_^2^ + 2*F*_c_^2^)/3
*S* = 1.10	(Δ/σ)_max_ = 0.001
2341 reflections	Δρ_max_ = 0.43 e Å^−^^3^
160 parameters	Δρ_min_ = −0.20 e Å^−^^3^
0 restraints	Absolute structure: Flack (1983), 952 Friedel pairs
Primary atom site location: structure-invariant direct methods	Flack parameter: 0.04 (5)

### Special details

**Table:**

Experimental. Crystallized from acetonitrile solution
Geometry. All e.s.d.'s (except the e.s.d. in the dihedral angle between two l.s. planes) are estimated using the full covariance matrix. The cell e.s.d.'s are taken into account individually in the estimation of e.s.d.'s in distances, angles and torsion angles; correlations between e.s.d.'s in cell parameters are only used when they are defined by crystal symmetry. An approximate (isotropic) treatment of cell e.s.d.'s is used for estimating e.s.d.'s involving l.s. planes.
Refinement. Data were collected by measuring three sets of exposures with the detector set at 2θ = 29°, crystal-to-detector distance 6.00 cm. Refinement of *F*^2^ against ALL reflections.

### Fractional atomic coordinates and isotropic or equivalent isotropic displacement parameters (Å^2^)

**Table:**

	*x*	*y*	*z*	*U*_iso_*/*U*_eq_	
Cl1	0.76710 (6)	1.02347 (3)	0.53880 (3)	0.02383 (10)	
Cl2	0.76768 (6)	0.86335 (3)	0.33094 (3)	0.02003 (10)	
O1	0.89020 (18)	0.30003 (10)	0.51020 (9)	0.0219 (2)	
O2	0.7597 (2)	0.41878 (9)	0.38886 (8)	0.0258 (3)	
C1	0.9599 (2)	0.30985 (14)	0.61831 (12)	0.0195 (3)	
H11	1.095 (3)	0.3243 (17)	0.6145 (14)	0.023*	
H12	0.928 (3)	0.2377 (17)	0.6515 (15)	0.023*	
C2	0.8628 (2)	0.41790 (14)	0.66439 (13)	0.0162 (3)	
H21	0.921 (2)	0.4618 (19)	0.7169 (15)	0.019*	
C3	0.6531 (2)	0.41869 (15)	0.65659 (14)	0.0183 (3)	
H31	0.586 (3)	0.469 (2)	0.6993 (15)	0.022*	
H32	0.590 (3)	0.3471 (17)	0.6365 (14)	0.022*	
C4	0.7718 (2)	0.48380 (12)	0.57217 (10)	0.0153 (3)	
C5	0.8034 (2)	0.40360 (13)	0.47933 (12)	0.0191 (3)	
C6	0.7654 (2)	0.61725 (12)	0.56086 (11)	0.0154 (3)	
C7	0.7598 (2)	0.68907 (12)	0.65134 (10)	0.0174 (3)	
H71	0.756 (3)	0.6558 (15)	0.7143 (14)	0.021*	
C8	0.7586 (2)	0.81285 (12)	0.64365 (11)	0.0183 (3)	
H81	0.746 (3)	0.8709 (14)	0.7047 (13)	0.022*	
C9	0.7631 (2)	0.86805 (11)	0.54561 (11)	0.0167 (3)	
C10	0.7664 (2)	0.79786 (12)	0.45517 (10)	0.0155 (3)	
C11	0.7674 (2)	0.67269 (12)	0.46254 (11)	0.0156 (3)	
H111	0.776 (3)	0.6262 (14)	0.4035 (14)	0.019*	

### Atomic displacement parameters (Å^2^)

**Table:**

	*U*^11^	*U*^22^	*U*^33^	*U*^12^	*U*^13^	*U*^23^
Cl1	0.0310 (2)	0.01352 (15)	0.02696 (19)	0.00112 (17)	−0.00448 (17)	0.00012 (12)
Cl2	0.02456 (18)	0.01897 (16)	0.01656 (16)	−0.00227 (16)	−0.00294 (15)	0.00519 (12)
O1	0.0341 (6)	0.0156 (5)	0.0160 (5)	−0.0008 (5)	0.0016 (5)	−0.0023 (4)
O2	0.0423 (7)	0.0211 (5)	0.0141 (5)	−0.0054 (6)	−0.0031 (6)	−0.0004 (4)
C1	0.0258 (8)	0.0161 (7)	0.0165 (7)	0.0021 (6)	0.0009 (6)	0.0023 (6)
C2	0.0203 (7)	0.0157 (7)	0.0126 (7)	0.0001 (6)	−0.0008 (6)	0.0010 (6)
C3	0.0216 (7)	0.0158 (7)	0.0175 (8)	−0.0008 (6)	0.0022 (6)	0.0030 (6)
C4	0.0186 (7)	0.0153 (6)	0.0120 (6)	−0.0015 (6)	−0.0015 (5)	0.0013 (5)
C5	0.0255 (8)	0.0142 (6)	0.0176 (7)	−0.0050 (6)	0.0003 (6)	−0.0004 (5)
C6	0.0142 (6)	0.0156 (6)	0.0165 (6)	−0.0012 (6)	−0.0012 (6)	0.0015 (5)
C7	0.0194 (7)	0.0192 (6)	0.0137 (6)	−0.0003 (7)	−0.0009 (6)	0.0018 (5)
C8	0.0179 (7)	0.0194 (6)	0.0176 (6)	0.0010 (7)	−0.0016 (6)	−0.0029 (5)
C9	0.0157 (6)	0.0129 (6)	0.0215 (7)	−0.0006 (6)	−0.0034 (6)	0.0003 (5)
C10	0.0140 (7)	0.0171 (6)	0.0153 (6)	−0.0010 (6)	−0.0019 (6)	0.0045 (5)
C11	0.0154 (7)	0.0168 (6)	0.0144 (6)	−0.0012 (6)	−0.0010 (6)	0.0003 (5)

### Geometric parameters (Å, °)

**Table:**

Cl1—C9	1.7329 (13)	C3—H32	0.949 (19)
Cl2—C10	1.7353 (13)	C4—C6	1.4934 (18)
O1—C5	1.3631 (19)	C4—C5	1.4940 (19)
O1—C1	1.4602 (19)	C6—C11	1.3909 (18)
O2—C5	1.1996 (18)	C6—C7	1.3988 (19)
C1—C2	1.503 (2)	C7—C8	1.3817 (19)
C1—H11	0.97 (2)	C7—H71	0.880 (17)
C1—H12	0.93 (2)	C8—C9	1.3868 (19)
C2—C3	1.484 (2)	C8—H81	1.012 (17)
C2—C4	1.522 (2)	C9—C10	1.3877 (19)
C2—H21	0.92 (2)	C10—C11	1.3968 (19)
C3—C4	1.541 (2)	C11—H111	0.912 (17)
C3—H31	0.91 (2)		
			
C5—O1—C1	110.94 (12)	C5—C4—C3	110.31 (12)
O1—C1—C2	105.73 (13)	C2—C4—C3	57.96 (9)
O1—C1—H11	107.3 (11)	O2—C5—O1	120.62 (14)
C2—C1—H11	109.6 (12)	O2—C5—C4	129.07 (14)
O1—C1—H12	106.0 (12)	O1—C5—C4	110.28 (13)
C2—C1—H12	113.7 (11)	C11—C6—C7	118.77 (12)
H11—C1—H12	113.8 (16)	C11—C6—C4	121.82 (12)
C3—C2—C1	115.72 (15)	C7—C6—C4	119.40 (12)
C3—C2—C4	61.65 (11)	C8—C7—C6	120.84 (13)
C1—C2—C4	106.25 (13)	C8—C7—H71	118.9 (11)
C3—C2—H21	119.1 (11)	C6—C7—H71	120.3 (11)
C1—C2—H21	120.2 (12)	C7—C8—C9	120.34 (12)
C4—C2—H21	119.1 (13)	C7—C8—H81	125.7 (9)
C2—C3—C4	60.40 (11)	C9—C8—H81	113.8 (9)
C2—C3—H31	118.9 (12)	C8—C9—C10	119.42 (12)
C4—C3—H31	113.9 (13)	C8—C9—Cl1	119.18 (10)
C2—C3—H32	118.9 (11)	C10—C9—Cl1	121.40 (10)
C4—C3—H32	117.8 (11)	C9—C10—C11	120.45 (12)
H31—C3—H32	115.4 (18)	C9—C10—Cl2	120.87 (10)
C6—C4—C5	121.57 (12)	C11—C10—Cl2	118.68 (10)
C6—C4—C2	124.48 (12)	C6—C11—C10	120.18 (13)
C5—C4—C2	104.70 (11)	C6—C11—H111	118.9 (10)
C6—C4—C3	121.22 (12)	C10—C11—H111	120.8 (10)
			
C1—C2—C4—C5	5.85 (15)	C3—C4—C5—O1	−57.45 (16)
O1—C1—C2—C4	−12.38 (16)	C2—C4—C6—C11	148.81 (15)
C2—C4—C5—O1	3.36 (16)	C3—C4—C6—C11	−140.85 (15)
C5—C4—C6—C11	7.2 (2)	C5—C4—C6—C7	−171.84 (15)
C5—O1—C1—C2	15.19 (17)	C2—C4—C6—C7	−30.3 (2)
O1—C1—C2—C3	53.50 (19)	C3—C4—C6—C7	40.1 (2)
C1—C2—C3—C4	−95.34 (15)	C11—C6—C7—C8	−0.7 (3)
C3—C2—C4—C6	108.17 (17)	C4—C6—C7—C8	178.38 (15)
C1—C2—C4—C6	−140.96 (15)	C6—C7—C8—C9	−0.1 (3)
C3—C2—C4—C5	−105.02 (14)	C7—C8—C9—C10	0.8 (3)
C1—C2—C4—C5	5.85 (15)	C7—C8—C9—Cl1	−178.46 (14)
C1—C2—C4—C3	110.87 (16)	C8—C9—C10—C11	−0.7 (2)
C2—C3—C4—C6	−113.68 (15)	Cl1—C9—C10—C11	178.55 (12)
C2—C3—C4—C5	95.02 (14)	C8—C9—C10—Cl2	178.79 (13)
C1—O1—C5—O2	169.94 (16)	Cl1—C9—C10—Cl2	−1.94 (19)
C1—O1—C5—C4	−11.76 (17)	C7—C6—C11—C10	0.8 (2)
C6—C4—C5—O2	−30.5 (3)	C4—C6—C11—C10	−178.25 (14)
C2—C4—C5—O2	−178.54 (17)	C9—C10—C11—C6	−0.1 (2)
C3—C4—C5—O2	120.66 (19)	Cl2—C10—C11—C6	−179.64 (12)
C6—C4—C5—O1	151.37 (14)		

### Hydrogen-bond geometry (Å, °)

**Table:**

*D*—H···*A*	*D*—H	H···*A*	*D*···*A*	*D*—H···*A*
C7—H71···O2^i^	0.880 (18)	2.366 (18)	3.2443 (16)	175.6 (16)

Symmetry codes: (i) −*x*+3/2, −*y*+1, *z*+1/2.
